# What makes a fang? Phylogenetic and ecological controls on tooth evolution in rear-fanged snakes

**DOI:** 10.1186/s12862-020-01645-0

**Published:** 2020-07-09

**Authors:** Erin P. Westeen, Andrew M. Durso, Michael C. Grundler, Daniel L. Rabosky, Alison R. Davis Rabosky

**Affiliations:** 1 Department of Environmental Science, Policy, and Management & Museum of Vertebrate Zoology, University of California, Berkeley, CA USA; 2 Department of Ecology and Evolutionary Biology & Museum of Zoology, University of Michigan, Ann Arbor, MI USA; 3 Department of Biological Sciences, Florida Gulf Coast University, Ft. Myers, FL USA

**Keywords:** Macroevolution, Morphology, Computed tomography, Evolutionary ecology, Dietary ecology, Dentition, Squamate reptiles

## Abstract

**Background:**

Fangs are a putative key innovation that revolutionized prey capture and feeding in snakes, and – along with their associated venom phenotypes – have made snakes perhaps the most medically-significant vertebrate animals. Three snake clades are known for their forward-positioned fangs, and these clades (Elapidae, Viperidae, and Atractaspidinae) contain the majority of snakes that are traditionally considered venomous. However, many other snakes are “rear-fanged”: they possess potentially venom-delivering teeth situated at the rear end of the upper jaw. Quantification of fang phenotypes – and especially those of rear-fanged species – has proved challenging or impossible owing to the small size and relative rarity of many such snakes. Consequently, it has been difficult to understand the evolutionary history of both venom and prey-capture strategies across extant snakes. We quantified variation in the dentition of 145 colubriform (“advanced”) snake species using microCT scanning and compared dental characters with ecological data on species’ diet and prey capture method(s) to understand broader patterns in snake fang evolution.

**Results:**

Dental traits such as maxilla length, tooth number, and fang size show strong phylogenetic signal across Colubriformes. We find extreme heterogeneity and evolutionary lability in the rear-fanged phenotype in colubrid (colubrine, dipsadine, and natricine lineages) and lamprophiid snakes, in contrast to relative uniformity in the front fanged phenotypes of other groups (vipers and, to a lesser extent, elapids). Fang size and position are correlated with venom-use in vipers, elapids, and colubrid snakes, with the latter group shifting fangs anteriorly by shortening the entire maxillary bone. We find that maxilla length and tooth number may also be correlated with the evolution of dietary specialization. Finally, an ancestral state reconstruction suggests that fang loss is a recurring phenomenon in colubrid snakes, likely accompanied by shifts in diet and prey capture mode.

**Conclusions:**

Our study provides a framework for quantifying the complex morphologies associated with venom use in snakes. Our results suggest that fang phenotypes, and particularly the rear-fanged phenotype, in snakes are both diverse and labile, facilitating a wide range of ecological strategies and contributing to spectacular radiations of these organisms in tropical and subtropical biomes worldwide.

## Background

Organismal morphology is constrained by phylogenetic history and shaped by novel selection pressures [1]. In snakes, variation in fang types is generally assumed to result from a diversity of ecological strategies as well as developmental constraints. The evolution of a venom delivery system at the base of the superclade of so-called “advanced” snakes (Colubriformes as in [2]) was a key innovation that may have contributed to the diversification of the group [3–6]. Colubriformes [2] comprises over 85% of extant snake diversity (> 3000 of > 3700 species), and includes all venomous snakes [7, 8]. No longer confined to constriction as the major method of subduing large prey, colubriform snakes were able to decouple the feeding and locomotor apparatuses [4, 9]. Subsequently, a variety of dental specializations evolved to facilitate the capture and consumption of diverse prey. These specializations, in turn, may have facilitated the global diversification of snakes, which are far more species-rich than any other comparable clade of squamate reptiles [10].

The teeth associated with venom delivery in snakes (e.g., fangs) occur on the upper jawbone known as the maxilla, which is distinct from the additional tooth-bearing bones (palatine, pterygoid) that comprise the upper palate. Colubriform maxillary dentition is highly variable, and has been used to estimate phylogenetic relationships among snake lineages [11–13], and to describe and identify particular species [14, 15]. The traditional classification system divides fangs into three main categories [14, 16]. The solenoglyphous fangs of vipers sit on a reduced and highly mobile maxillary bone [17, 18]. They are tubular with a smooth surface, and possess two orifices, one at either end, through which the venom enters and exits [19, 20]. The solenoglyphous condition (e.g., long and mobile “front” fangs) is probably what most laypersons have in mind when they think of venomous snakes and their teeth. A similar fang type evolved independently in some genera of African “mole vipers” (such as *Atractaspis,* which has a reduced but rotatable maxillary bone with a single large fang), though other Atractaspidines reveal various numbers of maxillary teeth [21–23]. In comparison, the proteroglyphous fangs of elapids – also “front-fanged” – are less mobile, significantly shorter, and sit on a reduced maxillary bone which may bear additional teeth posterior to the fang (as in the cobras; *Naja)* [12, 24, 25]. Lastly, the opisthoglyphous fangs (“rear fangs”) of species in a number of other colubriform lineages (e.g., Colubridae (as in [10], containing colubrine, dipsadine and natricine lineages), homolapsid, and lamprophiid snakes, hereafter referred to as non-elapid non-viperid (NE/NV) colubriforms) are located on the posterior of the maxillary bone and are grooved, rather than hollow [26]. In comparison to viperid and elapid fangs, relatively little is known about the opisthoglyphous condition, reflected in widespread disagreement over what exactly constitutes a “rear-fanged” snake [14, 27–30]. Recent discoveries have shown that front and rear fangs are homologous, with both front-fanged phenotypes (solenoglyphous vipers; proteroglyphous elapids) evolving independently from a rear-fanged ancestor [12, 27, 31, 32]. Though the folding fangs of vipers are often described as “perfect weapons” [33], the rifled-orifices on the fixed fangs of spitting cobras [34] and deep grooves on the rear fangs of boomslangs [35] reveal that there are multiple successful fang phenotypes.

The diversity of maxillary dentition across colubriform snakes represents one component of a suite of adaptations to the capture of diverse prey types [36, 37]. Constriction arose early in the evolutionary history of snakes, and indeed most booids and pythonoids – neither of which are Colubriformes – employ it as their primary method of prey subjugation [12, 38]. With the evolution of independent tooth-bearing bones in Macrostomatan (‘large-gaped’) snakes, the inner tooth rows (palatine and pterygoid) were found to be suitable for jaw walking over prey, thereby leaving the outer (maxillary) teeth free for specialization [9, 39, 40]. The evolution of venom and subsequently of fangs to deliver it allowed snakes to take fast-moving prey via ambush predation [41]. Vipers, for example, will typically strike a prey animal with erect fangs, wait for the venom to incapacitate it, and then locate and consume the animal [42, 43].

However, fangs are but one of many fascinating maxillary morphologies. Non-elapid/ non-viperid (NE/NV) colubriform snakes reveal a stunning variety of adaptations for capturing prey and feeding, including: the reduction of maxillary tooth size and number as an adaptation for oophagy (*Dasypeltis,* [44]); hinged teeth for durophagy (*Lycophidion,* [45]); recurved, striated teeth for capturing fish and amphibians (*Helicops, Hydrodynastes,* [46]); and enlarged maxillary and dentary teeth for extracting snails from their shells (*Dipsas,* [47]). In addition, medically-significant venoms are known from a number of NE/NV colubriform species. For example, African snakes in the genera *Dispholidus* (boomslang) and *Thelotornis* (savannah twigsnake) possess elongate and deeply grooved fangs; their venom is used to subdue lizards and other fast moving prey, and can be lethal to humans [48]. The enlarged but ungrooved rear fangs of the natricine *Rhabdophis tigrinus*, an amphibian specialist, have also caused human fatalities [22]. Despite intense study on a select few NE/NV colubriform species, the dental phenotypes and associated ecological significance has not been quantified for the vast majority of species in the group.

Here, we attempt to disentangle the contributions of evolutionary history and trophic ecology to dental morphology across colubriform snakes. We used microCT scanning to quantify maxillary dentition across 145 snake species and characterized the evolution of both morphological and ecological traits in a phylogenetic framework. Specifically, we sought to answer the following questions: (1) How do different dental traits co-vary across colubriform snakes, and within the opisthoglyphous/aglyphous colubrid lineages Colubrinae, Dipsadinae and Natricinae (2) How does dentition vary with different diets and methods of prey subjugation, and (3) is there evidence for fang loss across colubriform snakes?

## Results

### Computed tomography and trait measurements

**Fig. 1:**
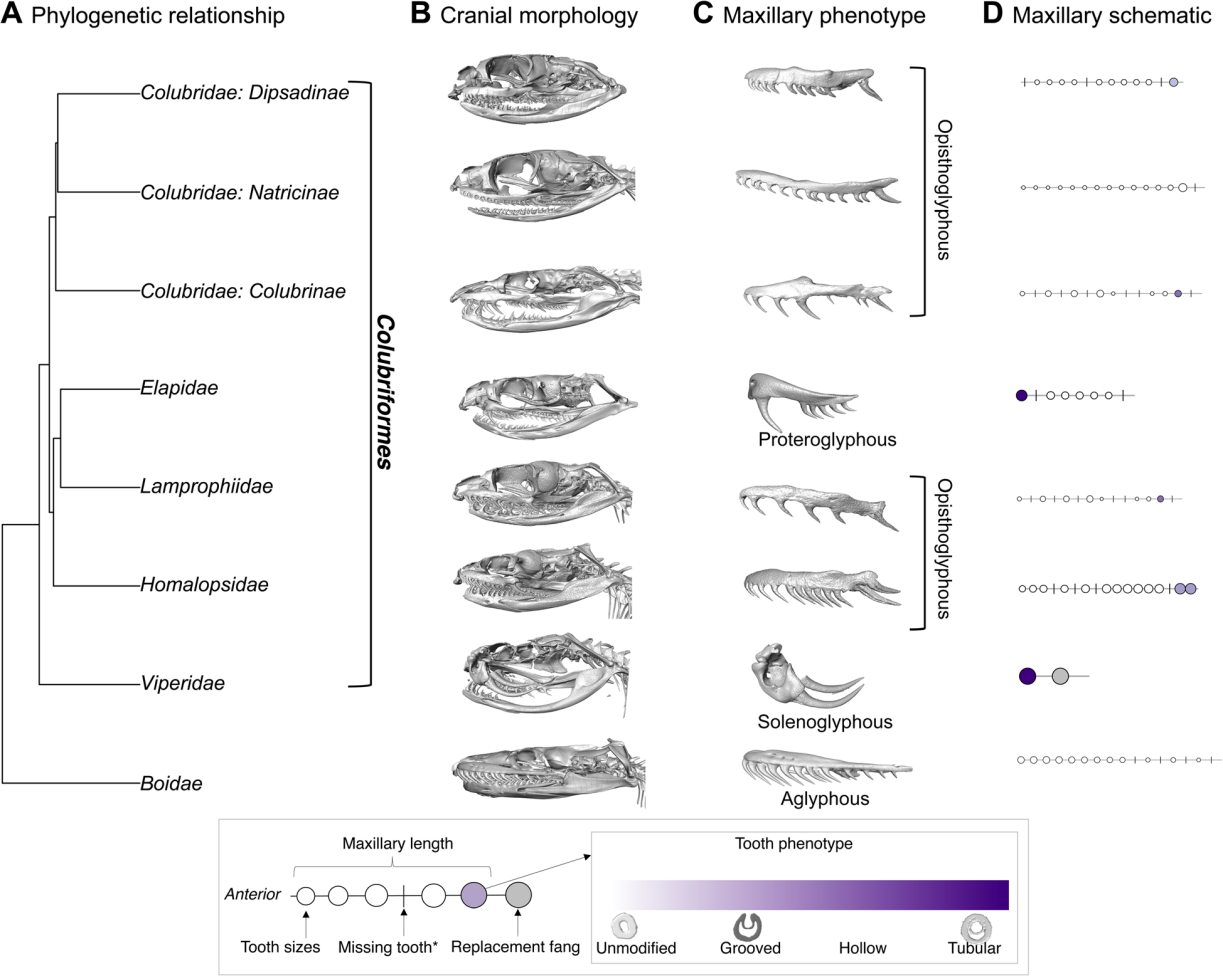
**a** Pruned phylogenetic tree depicting relationships between major snake families in this study. Colubriformes is a clade that includes all fanged snakes. **b** Cranial morphology for a single representative species from each family. **c** Segmented maxillary bones (which hold fangs, if present) from (**b**) reveal diversity in maxillary and dental morphology across families. Viperidae shows long, forward-positioned and rotatable (solenoglyphous) fangs. Forward-positioned but fixed (proteroglyphous) fangs are shown on the Elapid skull. A variety of rear-fanged (opisthoglyphous) phenotypes with different combinations of enlargement and grooving are shown on lamprophiid and colubrid representatives. **d** Schematic representation of average maxillary morphology, where line length represents maxillary length, teeth are shown as circles scaled by size, and tooth phenotype is represented by circle color. Circles represent rank-order of teeth on maxillary bone, rather than position, for simplicity. * “missing tooth” represents a tooth that was missing in the specimen as inferred from examination of sockets on maxillary bone, not a gap in tooth distribution

We microCT scanned and quantified morphological data for 145 species spanning 10 families and three colubrid subfamilies (Fig. 1). All specimens were scanned using high-resolution industrial CT scanners. We then segmented skull elements from images and generated corresponding surface renditions for each specimen. All image stacks, resulting models, and associated metadata are publicly available on MorphoSource. Technical details relating to scanning and image reconstruction are given in the Methods. Using the surface renditions we measured the following traits: length of the maxillary bone, number of teeth per tooth bearing bone (maxillary, palatine, pterygoid, dentary), length of each maxillary tooth, and groove dimensions (length, width, depth) of all grooved maxillary teeth (Supplementary Fig. 1). From these metrics, we derived fang position and size for rear-fanged snakes. We quantified fang size in rear-fanged snakes by modeling tooth length as a function of tooth position for the anterior maxillary teeth, and then calculating the residual between predicted and actual tooth lengths to assess the relative enlargement or reduction of the posterior teeth.

### Phylogenetic signal and covariance of dental traits

**Fig. 2:**
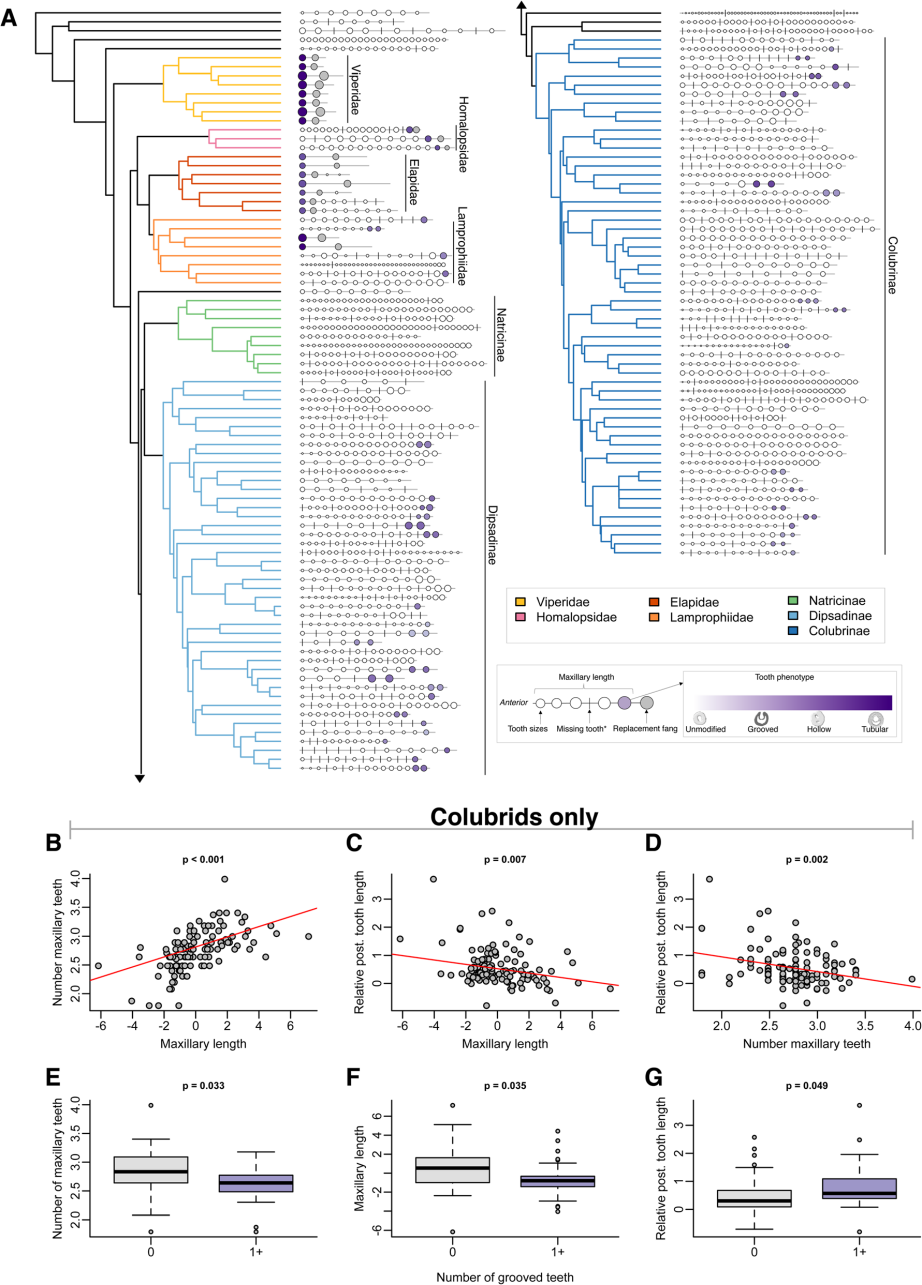
**a** Visualization of dental phenotypes across 145 snake species examined in this study, with Pyron et al. (2013) phylogeny for reference. Maxillary and dental traits are presented at the tips of the tree using the same schematic as in Fig. 1d. Each schematic represents a composite of the maxillary bones from one or more specimens. Note disparity in dental traits in non-front fanged lineages Colubridae and Lamprophiidae, compared to Viperidae, Elapidae. **b** - **d** Relationships between continuous maxillary traits for colubrid snakes only. Maxillary length and tooth length are given as residuals from linear regression of the corresponding trait against cranium length. **b** Positive relationship between maxillary length and number of maxillary teeth. **c**. Negative relationship between maxillary length and relative posterior tooth length (RPTL), a colubrid-specific measure of fang length. **d** Negative relationship between number of maxillary teeth and RPTL. **e** – **g** Boxplots of continuous traits grouped by posterior tooth phenotypes (0 grooved teeth vs. 1+ grooved teeth) for colubrids only. **e** Snakes with one or more grooved fang(s) have fewer maxillary teeth total. **f** Snakes with one or more grooved teeth have shorter maxillary bones. G. Snakes with one or more grooved teeth have larger fangs (RPTL)

Correcting dental traits for cranium size across all snakes using phylogenetic independent contrasts (PICs) revealed that maxillary length (F_1, 144_ = 281.1, R^2^ = .6589, *p* <  0.0001, slope = 0.467), position of largest tooth (F_1, 144_ = 7.567, R^2^ = 0.0433, *p* = 0.0067, slope = 0.008), and absolute fang size (F_1, 144_ = 154.5, R^2^ = .5142, p <  0.0001, slope = 0.058) scaled with cranium length, while number of maxillary teeth (F_1, 144_ = 0.742, R^2^ = − 0.0017, *p* = 0.3905, slope = 0.004), and groove width (F_1, 144_ = 1.369, R^2^ = 0.0025, *p* = 0.2439, slope = − 0.002) scaled independently of cranium size. Within NE/NV colubriforms, relative posterior maxillary tooth length scaled with cranium length (F_1, 112_ = 6.988, R^2^ = 0.0503, *p* = 0.009, slope = 0.008). After transforming trait values as needed, each dental trait showed strong evidence of phylogenetic signal (Table 1, Fig. 2a).

**Table 1:** Strong phylogenetic signal in each trait analyzed supports the notion that dental traits in snakes are controlled, to some extent, by evolutionary history, and provides justification for our use of comparative methods to understand correlations between traits

*Trait*	λ	*P-value*	*K*	*P-value*
Number of maxillary teeth	0.999	< 0.0001	1.281	0.001
Length of maxillary bone	0.999	< 0.0001	0.948	0.001
Position of largest tooth	0.812	< 0.0001	1.001	0.001
Groove width	0.917	< 0.0001	0.740	0.002
Fang size	0.999	< 0.0001	1.001	0.001
Relative posterior tooth length (colubrid only)	0.890	< 0.0001	0.735	0.002

To disentangle relationships between dental traits within colubrid (dipsadine, colubrine, natricine) snakes, we performed phylogenetic generalized models (PGLS) on colubrid snakes only. Number of maxillary teeth was significantly correlated with maxillary length (F_1, 113_ = 37.99, R^2^ = 0.245, *p* <  0.001, slope = 0.087) in colubrids. Relative posterior tooth length was negatively correlated with maxillary length (F_1, 113_ = 7.315, R^2^ = 0. 0525, *p* = 0.007, slope = − 0.077); species with shorter maxillary bones possess larger posterior teeth. The number of maxillary teeth was correlated with posterior tooth length (F_1, 113_ = 10.16, R^2^ = 0. 07435, *p* = 0.0018, slope = − 0.527), with species that possess fewer maxillary teeth having larger posterior maxillary teeth. Number of maxillary teeth also differs between species lacking vs. possessing grooves (t = 3.16, *p* = 0.033), with species possessing grooves having significantly fewer maxillary teeth by a factor of 0.795 teeth on average. Maxillary length differs between species that lack grooves vs. those that possess grooves (t = 3.293, *p* = 0.023), with those possessing grooves having significantly shorter maxillary bones relative to cranium size by a factor of 0.828 mm on average. Finally, species with one or more grooved teeth possess significantly larger posterior teeth (t = 2.856, *p* = 0.049) by a factor of 2.35 on average (rear fang length measured as residuals, see Methods for details). These relationships are summarized in Fig. 2b-g. The relationship between groove width and depth was non-significant (*p* = 0.269). Across families, snakes did not significantly vary in number of palatine teeth (F = 7.442, *p* = 0.216), nor pterygoid teeth (F = 1.27, *p* = 0.883), though there was much variation between individual species within these groups.

**Fig. 3:**
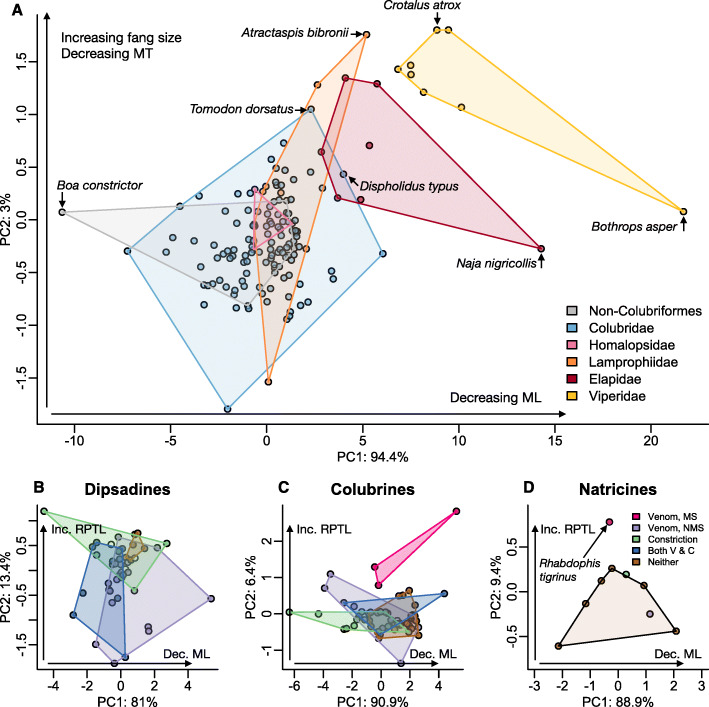
**a** Dental morphospace for all snakes in this study, visualized using the first two components of a phylogenetic PCA on univariate dental traits. Convex hulls group species’ points by family. Vipers occupy a distinct region of morphospace, with short maxillary bones and large fangs. Elapids are adjacent to vipers, but overlap in morphospace with some rear-fanged species within Homalopsidae and Colubridae. **b** – **d** First and second principal components from a pPCAfor colubrid subfamilies. Raw data for pPCA is the same as in A with the exception of one trait: fang length is replaced with relative posterior tooth length (RPTL) as a colubrid-specific metric of measuring the opisthoglyphous condition. Convex hulls in B-D group species by prey subjugation method: medically-significant (MS) venom, non-medically-significant (NMS) venom, constriction, both venom & constriction (V & C) and neither venom nor constriction. Across all subclades, maxillary length explains most variation in dentition. Venomous colubrids across all subfamilies but particularly B&C fill morphospace similar to that occupied by front fanged lineages in A. ML = maxillary length; MT = number of maxillary teeth; RPTL = relative posterior tooth length, a NE/NV colubriform specific measure of fang length

A principal components analysis of all snakes provides further evidence for phylogenetic conservatism in dental morphology (Fig. 3a, Table 2). Species generally cluster by family, with front-fanged lineages (viperids, elapids) occupying a distinct portion of morphospace, while aglyphous and opisthoglyphous lineages (colubrids, homalopsids, lamprophiids) overlap in morphospace (Fig. 3a).

**Table 2:** Principal component (PC) axis loadings for all snakes

*Trait*	PC1	PC2	PC3	PC4	PC5
Number of maxillary teeth	− 0.488	− 0.713	0.479	0.143	0.0385
Length of maxillary bone	−0.999	0.009	−0.007	− 0.002	0.000
Position of largest tooth	−0.025	0.045	0.653	−0.673	−0.342
Groove width	0.0501	0.0218	0.168	−0.480	0.858
Fang size	−0.042	0.855	0.487	0.164	0.041

### Prey subjugation mode and diet

The method of prey subjugation for species considered here was determined by literature search and categorized as: venom (medically-significant), venom (non-medically-significant), constriction only, venom and constriction (“both”), or neither venom nor constriction (“neither”).

**Fig. 4:**
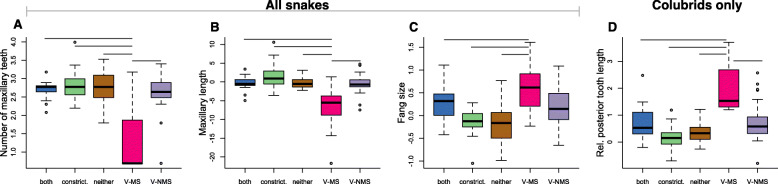
Boxplots showing dental traits that differ significantly when grouped by prey subjugation mode for all snakes (**a**-**c**) and colubrids (**d**). Each ANOVA revealed significant results (*p* < 0.05), and horizontal lines indicate significant differences between pairs of groups in post-hoc tests. “Both” = both venom & constriction, “constrict.” = constriction only, “neither” = neither venom nor constriction, “V-MS” = medically-significant venom, and “V-NMS” = non-medically significant venom. **a** Medically-significant venom users have fewer maxillary teeth than all other groups. **b** Medically-significant venom users have shorter maxillary bones than all other groups. **c** Medically-significant venom users have larger fangs than all other groups except non-medically-significant venom users. **d** In colubrids only, fang size (relative posterior tooth length) differs between medically-significant venom users and all other groups

Across all snakes, those utilizing different modes of prey subjugation differed in the number of maxillary teeth (Fig. 4a; F = 28.24, *p* <  0.001), and maxillary length (Fig. 4b; F = 22.66, p <  0.001) based on phylogenetic ANOVAs. Subsequent post-hoc pairwise tests revealed that medically-significant venom users differ in number of maxillary teeth from constrictors (t = − 9.209, *p* = 0.001; log-transformed tooth numbers, mean_V-MS_ = 1.323, mean_C_ = 2.814), species that use both venom and constriction (t = − 8.137, *p* = 0.001; mean_B_ = 2.68), species that do not use venom or constriction (t = − 5.53, *p* = 0.030; mean_N_ = 2.738), and non-medically-significant venom users (t = − 8.732, p = 0.001; mean_V-NMS_ = 2.605). Species that use medically-significant venom have shorter maxillae compared to species that use both venom and constriction (t = − 6.651, *p* = 0.0016; maxillae lengths presented as phylogenetic residuals, mean_V-MS_ = − 6.375, mean_B_ = − 0.386), constrictors (t = − 9.098, p = 0.001; mean_C_ = 1.578), those that use neither venom nor constriction (t = − 7.349, p = 0.001; mean_N_ = − 0.254), and those that use non-medically-significant venom (t = − 7.598, p = 0.0016; mean_V-NMS_ = − 0.537). In terms of fang size (Fig. 4c), medically-significant venom users differed from constrictors (t = 6.080, *p* = 0.0144; fang lengths presented as log-transformed phylogenetic residuals, mean_V-MS_ = 0.621, mean_C_ = − 0.127) and species using neither venom nor constriction (t = 6.886, *p* = 0.004; mean_N_ = − 0.161). Non-medically-significant venom users differed in fang size from species that use neither venom nor constriction (t = 4.100, *p* = 0.0135; mean_V-NMS_ = 0.206, mean_N_ = − 0.161). There is no significant correlation between prey subjugation mode and position of enlarged teeth (fang position; F = 6.77, *p* = 0.033, all post-hoc *p*-values > 0.05).

In analyses of colubrids only (excluding front-fanged vipers and elapids), we found significant differences in posterior tooth length between species using different prey subjugation modes (Fig. 4d). Medically-significant venom users differed from constrictors (t = 5.796, *p* = 0.001; posterior tooth lengths presented as phylogenetic residuals, mean_V-MS_ = 1.994, mean_C_ = 0.206), species that use constriction and venom (t = 3.955, *p* = 0.0483; mean_B_ = 0.751), species that use neither venom nor constriction (t = 5.458, p = 0.001), and non-medically-significant venom users (t = 4.235, *p* = 0.012; mean_N_ = 0.360). With regards to the presence of grooved posterior teeth, venom users differed from species using neither venom nor constriction (t = − 5.427, *p* = 0.0006); we did not detect a significant difference in presence of grooved teeth between venom users and constrictors at an alpha of 0.05 (t = 3.58, *p* = 0.112).

**Fig. 5:**
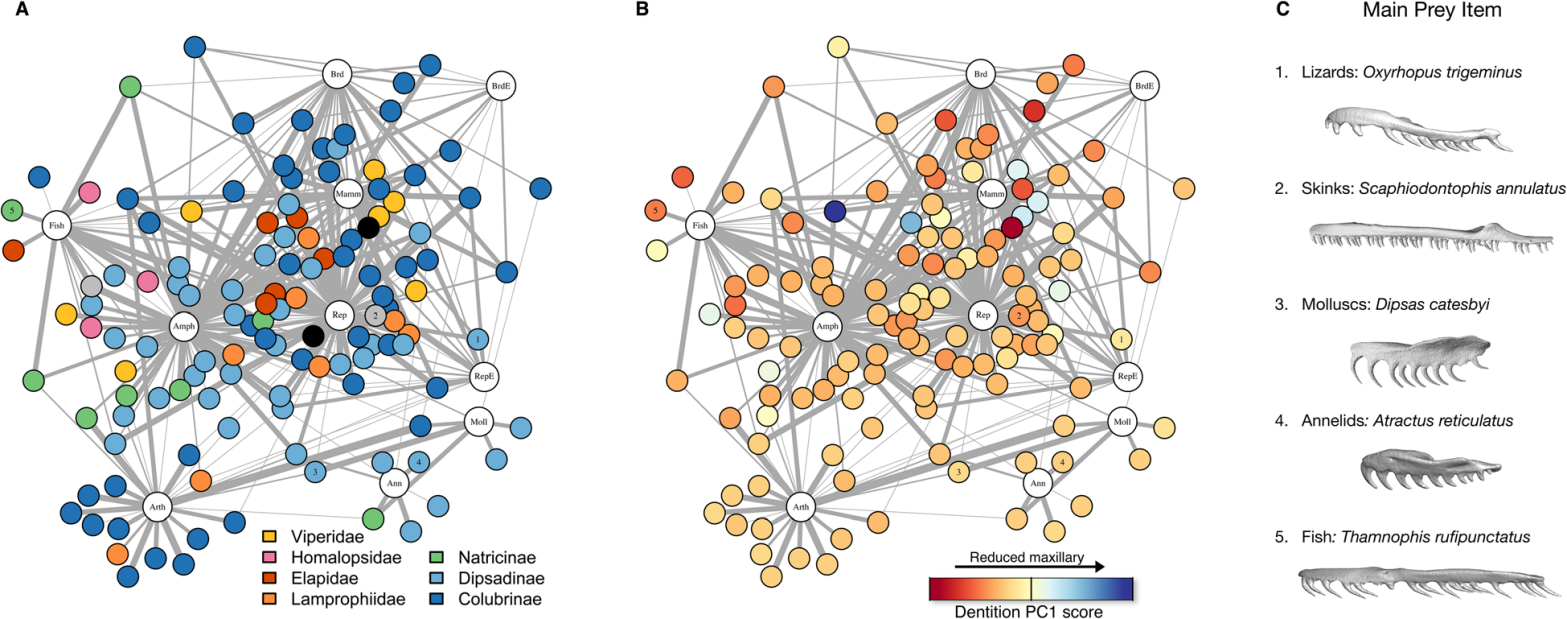
**a**-**b** Diet connectivity graphs for 124 species for which we quantified fang phenotype. Colored circles represent species (grey and black circles represent non-Colubriform species), and each species is connected to the diet items that it consumes (open circles with text labels). Line thickness represents relative importance of each diet item to the species. **a** Species’ nodes are colored by family. Colubrid (dipsadine, colubrine, natricine) snakes show connections to every diet category. **b** Colors correspond to the species’ score on dentition PC1, with higher scores being more viper-like (fewer maxillary teeth; blue) and lower scores representing species with many maxillary teeth (red). Reduced-maxilla phenotypes (blue) are clustered around vertebrate prey items, while intermediate phenotypes connect to nearly every diet category. Diet item abbreviations: BrdE = bird eggs, Brd = birds, Mamm = mammals, Amph = amphibians, Rep = reptiles, RepE = reptile eggs, Fish = fishes, Arth = arthropods, Ann = annelids, Moll = molluscs. **c** Representative NE/NV colubriform maxillary phenotypes with associated main prey items. Though skinks (lizards) are not included as a diet category in A & B, we show a representative to highlight the unique morphology associated with eating hard-bodied lizards

Diet data was gathered for 124 colubriform species via literature search. We constructed a diet matrix by coding the number of prey items recorded in each of 11 categories (reptiles, reptile eggs, birds, bird eggs, mammals, fishes, amphibians, annelids, arthropods, mollusks, and other) for each species. Using a phylogenetic Mantel test [49] we found no relationship between the diet matrix and the dentition matrix (r = − 0.03, *p* = 0.114). Phylogenetic ANOVAs between groups with different main prey items revealed that molluscivores possess fewer maxillary teeth compared to amphibian-eaters (t = − 3.34, *p* = 0.036; phylogenetic residuals maxillary tooth numbers, mean_molluscivores_ = 2.28, mean_amphib_ = 2.88) and fish-eaters (t = − 3.55, p = 0.036; mean_fish_ = 3.03), and shorter maxillary bones than piscivores (t = 3.219, p = 0.036; phylogenetic residual maxillary length, mean_mollusks_ = − 1.64, mean_fish_ = 1.736). All other dental traits did not differ significantly between diet groups. A diet network with each species connected to the prey items it consumes shows weak signal across families (Fig. 5a) and phenotypes (Fig. 5b).

### Ancestral state reconstruction/ fang loss

We performed two ancestral state reconstructions under the threshold model from quantitative genetics [50, 51]. We classified snake teeth in three states (unmodified, grooved, hollow) and four states (unmodified, grooved, hollow, tubular), with the four-state classification differentiating between the fangs of elapids (hollow) and vipers (tubular) to represent the independent origins of these fang phenotypes [31]. Both threshold-model analyses suggest multiple losses of posterior tooth grooving across colubriformes (Supplementary Figs. 2–3). These reconstructions suggest the colubriform common ancestor likely had a grooved fang, though this analysis is highly sensitive to the sampling design. We performed two additional analyses (three state and four state, as above) under a maximum likelihood framework using the ‘ace’ function in the R package ‘ape.’ For discrete characters, the likelihood values of a given node are calculated from the tip states of descendent lineages. We specified an equal rates model, in which transitions among all possible character states occur at the same rate (Supplementary Figs. 4–5). All four analyses gave highly congruent results (Supplementary Figs. 2–5) for several subclades that may have experienced the evolutionary loss of grooving. In one subclade of dipsadine snakes (*Pseudoboa*, *Clelia*, *Oxyrhopus* and *Hydrodynastes*) we found that the common ancestor likely possessed grooved maxillary teeth (p (grooved)_3-state model_ = 0.710, p (grooved)_4-state model_ = 0.940; marginal likelihood (grooved) _3-state_ model = 0.920, marginal likelihood (grooved) _4-state_ model = 0.955), but there is a reversal to the unmodified state in the species *Hydrodynastes gigas.* A similar scenario occurred in the clade containing *Thamnodynastes*, *Tomodon*, *Tachymenis*, and *Philodryas*, of which multiple species are known to use medically-significant venom (p (grooved)_3-state model_ = 0.510, p (grooved)_4-state model_ = 0.963; marginal likelihood (grooved) _3-state_ model = 0.914, marginal likelihood (grooved) _4-state_ model = 0.953); yet, the species *Helicops angulatus* and *Gomesophis brasiliensis* do not possess grooved teeth, suggesting another reversal.

## Discussion

Our results reveal extreme heterogeneity and lability in tooth number and size, as well as maxillary length, particularly in NE/NV colubriforms. By using continuous character coding, we find little support for the traditional notion of a canonical “rear-fanged” (opisthoglyphous) morphology. We show that prey capture method explains some variation in tooth number, tooth size and maxillary length across colubriform snakes, while dietary specialization may account for some variation in tooth number and maxilla length.

### How do dental traits co-vary across colubriform snakes and within colubrids (lineages Dipsadinae, Colubrinae and Natricinae)?

Broad differences in dentition across snakes are well established [5, 12, 26, 52], but patterns within NE/NV colubriforms are less well-resolved [3]. Here we find that mean numbers of palatine and pterygoid teeth do not differ across families, suggesting evidence for the hypothesis that Macrostomatan snakes use their inner tooth rows for the fairly conserved function of prey manipulation during consumption (‘pterygoid-walking’), while the outer tooth row was free to become more specialized for use in prey subjugation [39], but see [53]. Across all snakes, maxillary dentition is highly variable (Fig. 2a); yet strong phylogenetic signal indicates that it is generally conserved within families (Table 1; Fig. 3a). In NE/NV colubriforms, we find subclades revealing a gradient of phenotypes from long maxillary bones with many small teeth (e.g. *Scaphiodontophis annulatus, Grayia* spp.), to reduced maxillary bones with greatly enlarged, grooved posterior teeth (e.g. *Tomodon, Thamnodynastes*). Importantly, we show that colubrid snakes with larger posterior maxillary teeth have fewer maxillary teeth overall and shorter maxillae, and those with one or more grooved teeth have fewer maxillary teeth, shorter maxillae, and relatively larger posterior maxillary teeth (Fig. 2b-g). Enlarged, grooved posterior teeth on a reduced maxilla in opisthoglyphous species were described as early as 1896 [14] but have never been quantified due to difficulties in measuring small teeth. Using microCT scanning, we corroborate this relationship quantitatively for the first time. We further show that fang size of some NE/NV colubriforms, relative to cranium size, rivals that of their front-fanged counterparts (Figs. 2a and 3).

Grooving appears to be a highly labile characteristic across and within species (S. Figure 2-5); we do not find evidence for a relationship between groove dimensions (depth vs. width) within species. Selection for increased groove depth, eventually leading to an enclosed canal, remains a parsimonious explanation for the evolution of tubular fangs [54]. Though hollow fangs have evolved independently in vipers, elapids, and atractaspidines, other venom-using groups do not show this phenotype. Snakes with hollow fangs represent a small fraction of venomous species [32], which may be attributed to the cost of formation, or simple lack of variation in non front-fanged lineages.

### How does dentition vary with different methods of prey subjugation and prey items?

Across all snakes considered here, we find evidence that maxillary tooth number and maxillary length are correlated with prey subjugation mode, with medically-significant venom users possessing both fewer teeth and shorter maxillae compared to species that rely on alternative methods of prey capture (Fig. 4a-b). Medically-significant venom users have relatively larger fangs than other groups, though our sampling failed to detect a difference in fang length between medically-significant venom users and non medically-significant venom users (Fig. 4c), We suspect this result may be a consequence of oversampling colubrid snakes known to possess rear fangs, while sampling only a few representative vipers and elapids. In general, vipers and elapids with medically-significant venoms likely have larger fangs than most colubrids. However, the non-medically-significant category does includes a viper, *Causus rhombeatus,* that frequently inflicts non-serious bites [55–57]. Further, we found that neither group of venom users (medically-significant or non-medically-significant) differed from species that use both venom and constriction (Fig. 4c), a group that includes multiple elapids and at least one viper [58]. Additionally, many snakes have prey-specific venoms that do not affect humans: the medically significant framework is limited, but most data currently available regarding snake venoms is anthropocentric. Future studies will consider prey specificity when seeking to understand the relationships between dental morphology, venom composition, prey subjugation mode, and diet.

Further, fang size may be related to differential striking behaviors between groups [20]: while many vipers and some homalopsids typically strike and release prey, most rear-fanged snakes and elapids generally bite and hold [12, 59, 60]. Differences in fang length may reveal a tradeoff between striking and grasping capabilities [20]. The relationship between fang size, venom potency, and prey capture behavior requires further study across colubriform snakes.

In colubrids, relative posterior tooth length differed with mode of prey capture (Fig. 4d). Here, we see that species using medically-significant venom have larger fangs than all other groups. Consistent with expectations, species using venom (medically or non-medically-significant) were more likely to possess grooves on the posterior maxillary teeth relative to other groups. However, groups did not differ in maxillary length or number of teeth, despite a strong covariance between these traits across the family. Species using different prey subjugation modes did not differ in the position of enlarged teeth, suggesting that tooth enlargement may occur across the maxillary bone, unrelated to venom use [15]. While enlarged teeth are often described as “fangs,” enlargement could also be an adaptation for prey handling rather than venom delivery in many species [61].

Though posterior tooth enlargement and grooving are correlated with venom use in colubrids, both traits may not be necessary for venom delivery. In at least some species, venom is introduced by “chewing” once prey has been captured [59]. Enlargement of the posterior fangs may increase efficacy of venom delivery, but grooving alone may be sufficient for many species. Alternatively, some snakes possess enlarged teeth without grooves, and are known to use venom (e.g. *Rhabdophis, Xenodon*). Additionally, some snakes may possess enlarged posterior maxillary teeth that are so close together, they create an effective groove or single functional unit (e.g. *Diadophis punctatus* in our dataset) [24, 28, 46]: these snakes therefore possess enlargement and grooving together, but in a way not captured by traditional examinations of gross morphology. In colubrid snakes, it appears, there are many ways to make a fang.

Overall, we found weak signal between dental phenotype and diet (Fig. 5b). Diet explained differences in maxillary length and teeth in some colubriform groups, although this effect was driven in large part by species that consume gastropods. Specific adaptations such as enlarged dentary teeth and “handedness” (unequal numbers of maxillary teeth on the left and right side of the mouth) have been found in molluscivorous snakes, likely to aid in extraction of the organism from a hard shell [47, 62, 63]. We find gastropod eaters to possess shorter maxillary bones and fewer teeth, which may additionally aid in this endeavor. Piscivorous snakes are noted to have numerous and often posteriorly recurved maxillary teeth [46, 63]. Here we detect this signal, such that piscivores have more maxillary teeth compared to molluscivores. However, these ecological strategies may represent the “extremes,” with many snakes possessing both more generalist diets and intermediate maxillary phenotypes. These results must be interpreted in light of the fact that our understanding of snake trophic ecology is highly incomplete, owing to the difficulty of studying many species under field conditions [64]. While we can observe some general patterns in dentition across snake groups [46], morphological changes require further investigation as they relate to specific dietary shifts.

### Is there evidence for fang loss in colubriform groups?

Given that a single origin of snake fangs is likely and grooving appears to be a highly labile trait, the distribution of grooved teeth across NE/NV colubriform groups strongly suggests secondary losses in some groups. That fangs, and concordantly the use of venom, have been lost in many snakes has been assumed but never explicitly tested [3, 12, 65–67]. We find evidence for multiple losses of grooving, suggesting this phenomenon is common across colubriform snakes. Venom is costly to produce [68, 69], and therefore may be advantageous to lose in instances when it is not required for successful prey capture. However, a lack of fangs does not necessarily imply a lack of venom; studies of venom glands and proteins across species with wide-ranging dental phenotypes will further elucidate this relationship.

Loss of grooving in NE/NV colubriforms is likely related to species’ diet and method of prey capture. *Dipsas* species are specialized molluscivores and have dental adaptations for handling and processing these prey [70, 71]: the loss of grooving may have facilitated the evolution of other dental traits more suited to extracting snails from their shells. *Helicops angulatus* and *Gomesophis brasiliensis* are nested within a clade of species with grooved teeth, but have both lost grooves. *Helicops angulatus* is primarily a fish eater that subdues prey with both venom and constriction, while *Gomesophis brasiliensis* consumes invertebrates without venom or constriction. Further, many snakes in the colubrine rat snake clade primarily use constriction to subdue prey [72], and reveal no grooves on the posterior teeth. The venom glands in these species, as well as in specialized egg-eating sea snakes and molluscivores are severely atrophied [3], suggesting a loss of the venom delivery system entirely. These are but a few examples of what seems to be a widespread trend across the radiation; further studies will illuminate broader patterns of fang loss, and possibly, independent gains of fangs in colubriform snakes.

## Conclusions

Here, we have shown how evolutionary history and novel selection pressures have shaped the maxillary dentition of colubriform snakes. NE/NV colubriforms may be developmentally inhibited from shifting their fangs anteriorly as in elapids and vipers [31]: yet, we show that some colubrid snakes have adapted a similar but less extreme strategy to position their fangs more anteriorly by losing preceding teeth and shortening the maxilla. Yet other NE/NV colubriform species maintain the ancestral phenotype of elongated maxillary bones with unmodified teeth. We suggest that the variety of phenotypes observed in NE/NV colubriforms and especially colubrids should be viewed as a result of evolutionary lability rather than constraint, allowing for the evolution of diverse ecological specializations. In contrast, we propose that maxilla shortening in front fanged lineages was an irreversible step, leading these lineages to a single maxillary phenotype, respectively, and potentially limiting variation in trophic strategy. We show evidence for fang loss across Colubriformes, likely preceded by dietary shifts or the evolution of non-venom prey subjugation strategies. We emphasize that fang morphology is but one piece of the puzzle when considering the evolution of a venom delivery system. Studies of venom toxins and venom gland morphology, in concert with those of maxillary dentition, will provide a clearer picture of the evolution and diversification of venom delivery systems. Finally, we suggest that there are many ways to make a fang. The rear fangs of NE/NV colubriforms should not be viewed as an intermediate step on the path to an idealized, solenoglyphous fang, but rather a collection of unique phenotypes deserving of further study.

## Methods

### Sampling

We collected morphological data from preserved museum specimens maintained at the University of Michigan Museum of Zoology (UMMZ); three specimen models were taken from other museums (University of Florida, California Academy of Sciences) via MorphoSource.

As skull and tooth morphology are known to vary ontogenetically in snakes, we selected adult specimens for scanning. All specimens were scanned using high-resolution industrial CT scanners (uCT Scanco Medical; nanotom-s nanoCT with Phoenix Datos|× 2 Acquisition; Nikon XT H225ST, Dual tube system 180 kV and 225 kV. 2000 × 2000 detector). Voxel size varied with specimen size, and range between 12 and 40 μm. All image stacks, resulting models, and associated metadata are available online at MorphoSource [73]. We processed images in Avizo 9.2.0 3D software (FEI Company). Using the segmentation editor, we segmented skull elements from images and generated corresponding surface renditions for each specimen.

### Morphological measurements

From surface models, we recorded: number of teeth on each tooth bearing bone, the length of the maxillary bone, and skull dimensions including cranium length, width, and depth (Supplementary Fig. 1). Both the number of teeth present in the specimen as well as total number of teeth, inferred from examination of the bone and corresponding tooth sockets for missing teeth, were recorded. Because snakes often have one or more replacement teeth behind each functional tooth, but these teeth may not be fully developed or are non-functional, we measured only teeth ankylosed to the maxillary bone.

We then segmented the maxillary bones from each skull model. We measured the length of each maxillary tooth, from the base of the tooth where it is ankylosed to the maxillary bone, to the apical-most tip, using the 3D length tool. We recorded the putative position of all missing teeth using empty tooth sockets as guides. For specimens with grooved posterior maxillary teeth, we used the semi-landmarking tool to place 5 equidistant points from the base of the tooth to the apical-most point. At each of these points, we took measurements of tooth width, groove width, tooth depth, and groove depth. Groove length was also recorded. Measurements were repeated for each grooved tooth per specimen. From these, we derived the average relative groove dimensions (length, width, depth). We do not attempt to bin teeth into morphological or functional categories, but rather consider the number, position, enlargement, and grooving of the posterior maxillary teeth as continuous quantities to capture as much of the variation within these traits possible (Fig. 2a). We conducted all subsequent analyses in R version 3.4.4. For all analyses requiring phylogenetic information we used the phylogeny of Pyron et al. 2013.

Many NE/NV colubriform species show a trend of increasing enlargement in tooth size moving from anterior to posterior along the maxillary. To account for this background increase in tooth size in non-front fanged colubriforms, we fitted a linear model of tooth size ~ tooth position for all but the three posterior-most teeth on the maxillary using the “lm” function in the ‘stats’ package in R. We then used this model to predict the lengths of the three posterior-most maxillary teeth. We calculated the difference between the actual tooth lengths and predicted tooth lengths, and used the median residual as a metric of relative enlargement or reduction of the posterior teeth for that species.

We log-transformed the distribution of the number of maxillary teeth across species to achieve normality and confirmed by checking Q-Q plots. We then quantified correlations between each of our focal traits (number of maxillary teeth, maxillary length, position of largest tooth/teeth, groove width (if present), relative size of posterior maxillary teeth (NE/NV colubriforms) and absolute fang size (all snakes) with cranium size by performing phylogenetic independent contrasts (PICs) [74] between cranium length and morphological measurements. Traits found to correlate significantly with cranium size were regressed against cranium length, accounting for phylogeny, using the ‘phyl.resid’ function in phytools. We used residuals for all downstream analyses involving cranium size correlated traits.

### Phylogenetic signal and covariance of dental traits

We first tested for phylogenetic signal of all dental characters using the ‘phyl.sig’ function in phytools. We also performed a phylogenetic principal components analysis (pPCA) on the 5 focal dental traits (Tables 2, 3, 4 and 5), and grouped points by family to visually assess phylogenetic conservatism of dental traits (Fig. 3a). Then, we tested for correlations between all combinations of dental characters within a subset of NE/NV colubriform snakes (dipsadines, colubrines and natricines) with phylogenetic generalized linear models (PGLS), using the ‘pgls’ function in caper. We also tested the relationship between groove depth and groove width across all snakes that possessed one or more grooved teeth using PGLS. Next, we tested whether species that do or do not have grooved teeth differ in number of maxillary teeth and maxillary length using the ‘phylANOVA’ function in phytools. Finally, we explored whether colubriform snakes differ in numbers of teeth on other tooth bearing bones by family. We excluded families with only one specimen examined (Pareidae).

**Table 3:** Principal component (PC) axis loadings for dipsadine snakes

*Trait*	PC1	PC2	PC3	PC4	PC5
Number of maxillary teeth	−0.375	0.226	0.893	0.007	0.101
Length of maxillary bone	−0.999	−0.023	− 0.015	0.002	− 0.001
Position of largest tooth	0.068	−0.179	0.342	−0.673	0.595
Groove width	0.136	0.068	−0.200	0.893	0.371
Fang size	0.118	−0.991	0.055	−0.002	0.020

**Table 4:** Principal component (PC) axis loadings for colubrine snakes

*Trait*	PC1	PC2	PC3	PC4	PC5
Number of maxillary teeth	−0.585	− 0.235	−0.560	− 0.293	0.448
Length of maxillary bone	−0.999	0.028	0.004	0.000	−0.004
Position of largest tooth	0.054	0.151	−0.813	0.508	−0.212
Groove width	0.143	−0.013	−0.365	− 0.677	−0.622
Fang size	0.353	0.934	−0.012	−0.029	0.030

**Table 5:** Principal component (PC) axis loadings for natricine snakes

*Trait*	PC1	PC2	PC3	PC4
Number of maxillary teeth	−0.683	−0.309	0.606	0.263
Length of maxillary bone	−0.999	0.002	−0.015	−0.000
Position of largest tooth	−0.449	−0.692	0.510	−0.242
Fang size	0.099	−0.985	−0.133	0.025

### Prey subjugation mode and diet

We searched relevant literature for descriptions of prey subjugation behavior for each species. We categorized prey subjugation mode as one of five categories: venom (medically-significant), venom (not medically-significant), constriction, venom and constriction, or neither venom nor constriction.

We removed any species for which the method of prey capture could not be determined, resulting in 141 species analyzed. To examine the association between snakes’ method of prey capture and dentition, we tested for the effects of prey subjugation mode on univariate dental traits using phylogenetically corrected ANOVAs [75] with the ‘phylANOVA’ function in phytools. If significance was determined, we ran post-hoc tests between all groups. We ran these analyses across all snakes studied, as well as for colubrid (dipsadine, colubrine and natricine) snakes only.

We then ran independent pPCAs for Dipsadinae, Colubrinae and Natricinae: we substituted fang length in the prior pPCA with relative posterior tooth length (RPTL) as a NE/NV colubriform specific metric of fang length (Tables 3, 4 and 5, respectively). We grouped results by prey subjugation mode to assess how dentition varies by ecological strategy (Fig. 3b-d).

We surveyed the published literature for quantitative data on the diet contents of these species, resulting in data for 124 colubriform species. Quantitative data included any diet observation for which it was possible to determine the number of individual predators or prey involved. Thus, our database comprises a heterogeneous mixture of studies that includes observations from dissections of museum specimens as well as observations from chance encounters with free-ranging snakes caught in the act of consuming prey. We categorized diet observations in 11 prey categories as follows: reptiles, reptile eggs, birds, bird eggs, mammals, fishes, amphibians, annelids, arthropods, mollusks, and other. This categorization scheme allowed us to pool data from multiple sources. To visualize the variation in colubroid diet composition, we created a diet graph in which prey items and snake species are represented by vertices and trophic relationships as edges (Fig. 5). Line thickness indicates relative importance of each prey item to the species. We tested for a relationship between dentition and diet by first computing dissimilarity matrices between species for all dental traits (counts and linear measurements) and prey items (counts per diet category) using the ‘daisy’ function in the cluster package. This method creates pairwise distance matrices between all species pairs in both morphological and diet space. We then used a phylogenetic Mantel test with Euclidean distance, implemented with the function ‘phyloMantel’ in package evolqg. Finally, we computed the most commonly observed prey item per species. We ran phylogenetic ANOVAs to test for differences in dentition between groups on the basis of primary diet item, and post-hoc tests when significance was determined.

### Ancestral state reconstruction/fang loss

We estimated the ancestral fang phenotype for colubriform snake based on our morphological data. We classified species’ fang phenotype in two ways: a three-state scheme (unmodified, grooved, hollow) and a four-state scheme that differentiates within front fangs (unmodified, grooved, hollow (elapids, some atractaspidines), tubular (vipers). We performed two sets of analyses: (1) using the ‘ancthresh’ function in phytools, which uses Bayesian MCMC to estimate ancestral states for discrete characters under the threshold model from quantitative genetics (100,000 generations, 20,000 burn-in generations), and (2) using the ‘ace’ function in the ‘ape’ package, which uses maximum likelihood to calculate ancestral states. In the ‘ace’ analyses we specified an equal rates model, in which transitions among all character states occur at the same rate [50]. Though our relatively small sample set limits our ability to accurately reconstruct ancestral states, we focus on subclades for which we have thorough sampling to assess evidence for fang loss.

## Supplementary information

**Additional file 1: Fig. S1.** Sample skull model with measurements applied. Cranium length was measured from the tip of the premaxillary bone to the base of the quadrate bone. Each maxillary tooth was measured from the point of contact with the maxillary bone to the apical-most point (TL). All measurements were repeated three times each and the average value was used for subsequent analyses. **Fig. S2.** Ancestral state reconstruction of maxillary tooth phenotype in which fang phenotypes were coded as one of three states: unmodified, grooved, or hollow. We used the ‘ancthresh’ function in phytools which implements Bayesian MCMC to estimate ancestral states for discrete characters under the threshold model from quantitative genetics (100,000 generations, 20,000 burn-in generations). **Fig. S3.** The same analysis as S2 (‘ancthresh’ Bayesian MCMC estimation of ancestral states, 100,000 generations, 20,000 burn-in generations) was run with four possible fang states: unmodified, grooved, hollow (elapids, some lamprophiids), or tubular (vipers). Results between these two models (S2 and S3) are highly consistent, and both show likely reversals from the grooved state to the unmodified state (*Hydrodynastes gigas; Gomesophis brasiliensis* and *Helicops angulatus; Conopsis nasus*), though the posterior probability of fang loss in the NE/NV colubriform is higher in the three-state model. Whether front fangs are grouped into a single category, or treated as unique character states, it appears likely that rear fangs have been lost in NE/NV colubriforms on more than one occasion. **Fig. S4.** We reconstructed ancestral character states under a maximum likelihood framework using the ‘ace’ function in the R package ‘ape.’ For discrete characters, the likelihood values of a given node are calculated from the tip states of descendent lineages. We specified an equal rates model, in which transitions among all possible character states occur at the same rate. Here the results are shown for a scenario in which we classified fangs in 3 states (unmodified, grooved, hollow), as in S. Figure 2. **Fig. S5.** The same analysis as S4 is shown here for a four-state characterization of fangs. Results of all four models (S2-S5) for the nodes of interest are highly congruent (see main text). The main difference between ancThresh and ace outputs are that likelihood-based analyses (ace, S4 and S5) do not support the notion that the colubriform common ancestor possessed grooved fangs, based on our sampling.

## Data Availability

The microCT data supporting the conclusions of this article are available at Morphosource (https://www.morphosource.org/Detail/ProjectDetail/Show/project_id/374, https://www.morphosource.org/Detail/ProjectDetail/Show/project_id/490). Other data (morphological measurements, diet data, and prey subjugation notes) supporting the conclusions of this article are available on Dryad: Westeen, Erin et al. (2020), What makes a fang? Phylogenetic and ecological controls on tooth evolution in rear-fanged snakes, v2, UC Berkeley, Dataset, 10.6078/D17M5J.

## Abbreviations

CT: Computed tomography
NE/NV: Non-Elapid Non-Viperid
VMS: Venom, medically-significant
VNMS: Venom, non-medically-significant
RPTL: Relative posterior tooth length
