# Supplementary material for: What makes a fang? Phylogenetic and ecological controls on tooth evolution in rear-fanged snakes
Source: BMC Evol Biol. 2020 Jul 9;20:80. doi: 10.1186/s12862-020-01645-0 (PMC7346461; doi:10.1186/s12862-020-01645-0)
Supplement: Supplementary file 1 — Additional file 1: Fig. S1. Sample skull model with measurements applied. Cranium length was measured from the tip of the premaxillary bone to the base of the quadrate bone. Each maxillary tooth was measured from the point of contact with the maxillary bone to the apical-most point (TL). All measurements were repeated three times each and the average value was used for subsequent analyses. Fig. S2. Ancestral state reconstruction of maxillary tooth phenotype in which fang phenotypes were coded as one of three states: unmodified, grooved, or hollow. We used the ‘ancthresh’ function in phytools which implements Bayesian MCMC to estimate ancestral states for discrete characters under the threshold model from quantitative genetics (100,000 generations, 20,000 burn-in generations). Fig. S3. The same analysis as S2 (‘ancthresh’ Bayesian MCMC estimation of ancestral states, 100,000 generations, 20,000 burn-in generations) was run with four possible fang states: unmodified, grooved, hollow (elapids, some lamprophiids), or tubular (vipers). Results between these two models (S2 and S3) are highly consistent, and both show likely reversals from the grooved state to the unmodified state (Hydrodynastes gigas; Gomesophis brasiliensis and Helicops angulatus; Conopsis nasus), though the posterior probability of fang loss in the NE/NV colubriform is higher in the three-state model. Whether front fangs are grouped into a single category, or treated as unique character states, it appears likely that rear fangs have been lost in NE/NV colubriforms on more than one occasion. Fig. S4. We reconstructed ancestral character states under a maximum likelihood framework using the ‘ace’ function in the R package ‘ape.’ For discrete characters, the likelihood values of a given node are calculated from the tip states of descendent lineages. We specified an equal rates model, in which transitions among all possible character states occur at the same rate. Here the results are shown for a scenario i [file 12862_2020_1645_MOESM1_ESM.docx]

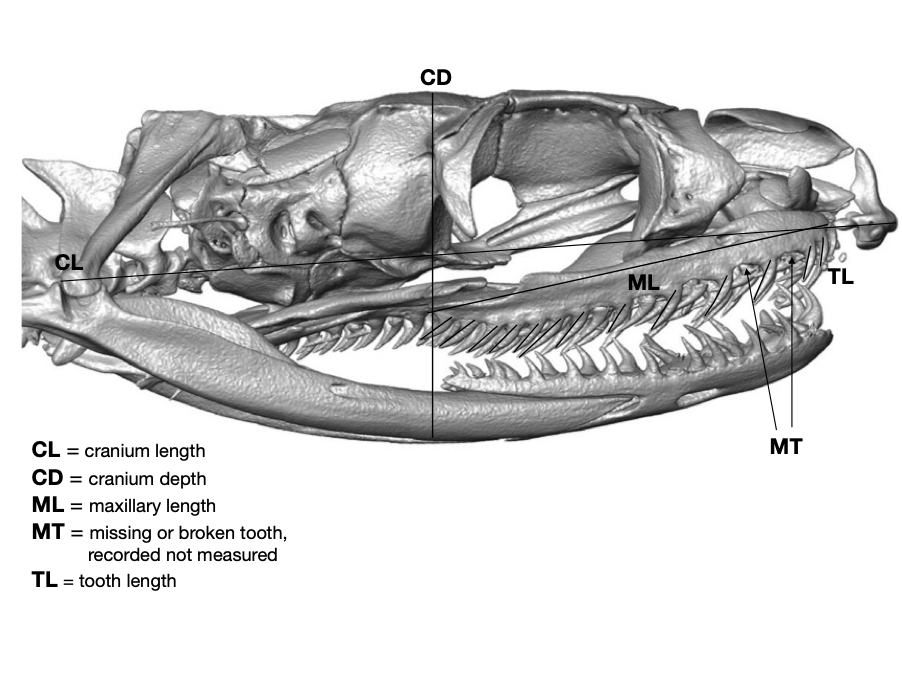


Fig. S1: Sample skull model with measurements applied. Cranium length was measured from the tip of the premaxillary bone to the base of the quadrate bone. Each maxillary tooth was measured from the point of contact with the maxillary bone to the apical-most point (TL). All measurements were repeated three times each and the average value was used for subsequent analyses.

Fig. S2: Ancestral state reconstruction of maxillary tooth phenotype in which fang phenotypes were coded as one of three states: unmodified, grooved, or hollow. We used the ‘ancthresh’ function in phytools which implements Bayesian MCMC to estimate ancestral states for discrete characters under the threshold model from quantitative genetics (100000 generations, 20000 burn-in generations).

Fig S3: The same analysis as S2 (‘ancthresh’ Bayesian MCMC estimation of ancestral states, 100000 generations, 20000 burn-in generations) was run with four possible fang states: unmodified, grooved, hollow (elapids, some lamprophiids), or tubular (vipers). Results between these two models (S2 and S3) are highly consistent, and both show likely reversals from the grooved state to the unmodified state (*Hydrodynastes gigas; Gomesophis brasiliensis* and *Helicops angulatus; Conopsis nasus*), though the posterior probability of fang loss in the NE/NV colubriform is higher in the three-state model. Whether front fangs are grouped into a single category, or treated as unique character states, it appears likely that rear fangs have been lost in NE/NV colubriforms on more than one occasion.


Fig S4: We reconstructed ancestral character states under a maximum likelihood framework using the ‘ace’ function in the R package ‘ape.’ For discrete characters, the likelihood values of a given node are calculated from the tip states of descendent lineages. We specified an equal rates model, in which transitions among all possible character states occur at the same rate. Here the results are shown for a scenario in which we classified fangs in 3 states (unmodified, grooved, hollow), as in S. Fig. 2.

Fig S5: The same analysis as S4 is shown here for a four-state characterization of fangs. Results of all four models (S2-S5) for the nodes of interest are highly congruent (see main text). The main difference between ancThresh and ace outputs are that likelihood-based analyses (ace, S4 and S5) do not support the notion that the colubriform common ancestor possessed grooved fangs, based on our sampling.
